# Discrepancy between the initial assessment of injury severity and post hoc determination of injury severity in patients with apparently mild traumatic brain injury: a retrospective multicenter cohort analysis

**DOI:** 10.1007/s00068-017-0861-z

**Published:** 2017-10-14

**Authors:** S. M. Bossers, K. M. Pol, E. P. A. Oude Ophuis, B. Jacobs, M. C. Visser, S. A. Loer, C. Boer, J. van der Naalt, P. Schober

**Affiliations:** 1 Department of Anesthesiology, VU University Amsterdam Medical Center, De Boelelaan 1117, 1081 HV Amsterdam, The Netherlands; 2 University of Groningen, University Medical Center Groningen, Department of Neurology, Hanzeplein 1, 9713 GZ Groningen, The Netherlands; 3 Department of Neurology, VU University Medical Center, De Boelelaan 1117, 1081 HV Amsterdam, The Netherlands

**Keywords:** Mild traumatic brain injury, Emergency medical service, Hospital, Decision support techniques, Prognosis, Risk factors

## Abstract

**Purpose:**

Traumatic brain injury (TBI) is a major cause of trauma-related visits to emergency departments (ED). Determination of monitoring requirements of patients with apparently mild TBI is challenging. Patients may turn out to be more severely injured than initially assumed, and failure to identify these patients constitutes a serious threat to patient safety. We, therefore, aimed to identify clinical risk factors for more severe injuries in patients with apparently mild TBI.

**Methods:**

In a retrospective cohort analysis performed at two level I trauma centers, 808 patients aged ≥ 16 presenting to the ED with head trauma and a Glasgow Coma Scale (GCS) score 13–15 who received a head CT scan were studied. Discrepancies between the initial TBI severity as determined by GCS and severity as determined post hoc by the Head Abbreviated Injury Score were assessed. Multiple logistic regression was used to identify risk factors of such discrepancies.

**Results:**

104 (12.9%) patients were more severely injured than initially classified. A GCS < 15 at presentation (GCS 13: OR 6.2, [95% CI 3.8–9.9]; GCS 14: OR 2.7, [2.0–3.7]), an SpO_2_ < 90% (OR 5.4, [1.2–23.4]), loss of consciousness (OR 2.3, [1.5–3.5]), absence of equal and reactive pupils (OR 2.1, [1.6–2.7]), transport by ambulance (OR 2.0, [1.7–2.4]), and use of anticoagulant drugs (OR 1.2, [1.1–1.3]) were independent risk factors of more severe injury.

**Conclusions:**

Six risk factors of more severe injury in patients presenting with apparently mild TBI were identified. Patients with any of these factors should be thoroughly monitored for signs of neurologic deterioration.

## Introduction

Traumatic brain injury (TBI) is one of the most common injuries in the western world [1], and represents an important cause of morbidity and mortality, especially in the first decades of life [2]. TBI severity is commonly estimated using the Glasgow Coma Scale (GCS) and classified as either mild (GCS score 13–15), moderate (GCS score 9–12), or severe (GCS score 3–8) [3–5]. Patients with moderate or severe TBI, who present with impaired consciousness and often have obvious external injuries, are usually easily identified as requiring prompt attention and intensive follow-up. In contrast, determination of monitoring requirements of patients with an initial presentation of mild TBI can be more challenging. Healthcare providers need to determine which patients require additional diagnostics and monitoring, and who can be discharged safely. Patients with apparently minor injuries at the first presentation may turn out to be more severely injured than initially assumed and/or assessed [6]. Failure to identify such patients during the initial assessment can result in under-treatment and constitutes a serious threat to patient safety. Additional insights in the relationship between clinical parameters that are readily available to the clinician at the first presentation and injury severity may be useful to identify patients at risk and to determine monitoring requirements. We, therefore, aimed to identify clinical risk factors for a discrepancy between the initial TBI severity as determined by GCS at the initial presentation and actual injury severity as determined post hoc in patients with apparently mild TBI.

## Methods

### Study design

The study is a retrospective cohort analysis performed at two level I trauma centers in the Netherlands, the VU University Medical Center Amsterdam (VUmc) and the University Medical Center Groningen (UMCG). The Institutional Review Board of the VUmc determined that formal approval of the study was not required and waived the need of informed consent.

### Patient population

All consecutive patients with a head trauma and who received a head CT scan between January and July 2011 in the VUmc and between January and December 2011 in the UMCG were screened for eligibility. Patients ≥ 16 years were included if they presented with mild TBI, defined by a head trauma with a GCS score of 13–15 at the initial presentation, and if the Head Abbreviated Injury Score (H-AIS) was available. Patients presenting to the emergency department more than 24 h after the trauma and patients who were directly transported to another hospital after the initial assessment were not included.

### Definition of discrepancy between injury severity at the initial presentation and actual injury severity

In line with common practice, we defined TBI severity at the initial assessment based on the first documented GCS score in the emergency room as described above. The Head Abbreviated Injury Score (H-AIS, version 1995, update 1998) was used as a second marker for injury severity [7]. This score is assigned in retrospect when all information is available, and is based on clinical signs and symptoms as well as pathologic-anatomical and radiologic findings [8]. Therefore, this scale cannot be used for the initial assessment, but it is useful for a post hoc determination of the actual severity. The H-AIS score ranges from 1 to 6 (minor injury to unsurvivable injury), and based on corresponding mortality rates of H-AIS and GCS, H-AIS 1–2 corresponds to mild TBI [9]. Discrepancies between the initial and post hoc classification of severity were assessed, and H-AIS scores > 2 suggest that the patient’s head injury was actually more severe than assumed during the initial assessment. The presence or absence of a discrepancy between the initial assessment of injury severity and post hoc determination of injury severity was coded as a binomial variable, which was subsequently used as dependent variable in the statistical analyses, as described in detail below.

### Data collection

Data were extracted using a standardized data collection protocol from digital and paper medical records, including demographic data, injury characteristics, mode of transport to hospital (transport by ambulance vs. self-referral by private or public transport), details of physical examination, vital parameters, laboratory and radiology examination results, as well as interventions, treatments, and measures of outcome at discharge. The cerebral CT scans were scored using the Marshall classification and the Rotterdam score [10, 11].

### Statistical analysis

Data were analyzed with STATA 13.1 (StataCorp, College Station, TX). Continuous data (e.g., age) showed significant deviations from the normal distribution. Therefore, continuous data, as well as ordinal data (e.g., GCS score), are shown as median with interquartile range; and nominal data (e.g., gender) are presented as percentages. Mann–Whitney U tests and Chi-square tests were used to test for differences in various parameters between patients who show and do not show a discrepancy between the initial injury severity and the injury severity as determined by the H-AIS score.

For multivariable analyses, multiple imputation was used to minimize bias due to missing data. A total of 100 data sets were imputed in which missing values were iteratively replaced using chained equations [12]. Subsequently, logistic regression, adjusted for clustering by hospital to account for the multicenter study design, was applied to the imputed data sets to address the relationship between predictor variables and discrepancies in injury severity while controlling for the effects of the other independent variables. Regression parameters were pooled across the imputed data sets to obtain an overall estimate, and Wald-test *p* values were used to address statistical significance.

For the model development, we used a modification of the stepwise variable selection approach described by Hosmer et al. [13]: in a first step, a full model including 16 potentially relevant clinical predictor variables that are readily assessable at presentation in the emergency department was selected. To obtain a parsimonious model, stepwise backward elimination of variables with a *p* value > 0.05 was performed, until all variables in the model showed a significant (*p* < 0.05) relationship with discrepancy in injury severity. Subsequently, all previously eliminated variables were re-entered one-by-one and were retained in the model if the *p* value provided evidence for an association with the outcome variable. This step was repeated until a final model (model A) was obtained in which all independent variables were significantly associated with the outcome variable. Goodness of fit was assessed using Hosmer–Lemeshow tests and Pearson’s chi-square goodness of fit tests.

Another logistic regression model (model B) was used to determine the independent effects of the clinical predictors from model A, after adjusting for trauma-related pathology on the cerebral CT scan.

## Results

A total of 808 patients matched the inclusion criteria. Of these, 498 patients were included in the VUmc and 310 in the UMCG. One hundred and four patients (12.9%, 95% CI 10.6–15.2%) showed a discrepancy between the initial severity based on the GCS at the first assessment and the actual severity as determined post hoc by the H-AIS score. Most of these patients had signs of trauma-related intracranial pathology on the initial CT scan; however, 13.4% of these patients had a normal scan. Table 1 summarizes crude demographic data as well as injury and outcome characteristics of patients with and without a discrepancy between the initial injury severity and the actual injury severity.

**Table 1:** Crude demographic data as well as injury and outcome characteristics of patients with and without a discrepancy between the initial assessment of injury severity and actual injury severity

	No discrepancy	Discrepancy	*p* value	% missing
*N*	704	104		0
Demographic characteristics				
Age (median years)	46 (26–68)	47 (29–65)	0.865	0
Gender (male%/female%)	57/43	73/27	0.002	0
Anamnestic characteristics				
Prevalence of anticoagulant use (%)	24.3	24.3	0.998	1.1
Loss of consciousness (%)	41.9	71.1	< 0.001	24.1
Amnesia (%)	48.5	78.9	< 0.001	10.8
Nausea (%)	22.0	24.2	0.634	5.7
Vomiting (%)	8.3	18.2	0.002	4.6
Headache (%)	35.2	37.8	0.658	37.0
PEARL (%)	96.4	91.4	0.018	1.5
Alcohol intoxication (%)	38.0	31.6	0.229	17.0
Drugs intoxication (%)	4.2	6.3	0.348	17.0
In-hospital characteristics				
Presented to hospital by EMS (%)	74.2	85.9	0.012	7.2
Admission during office hours (%)	27.5	25.0	0.592	0.7
GCS at ED admission (median)	15 (15–15)	14 (14–15)	< 0.001	0
GCS 15 (%)	79.3	49.0		
GCS 14 (%)	17.2	35.6		
GCS 13 (%)	3.6	15.4		
SBP on ED admission (median mmHg)	140 (122–155)	130 (117–150)	0.089	15.3
Hypotension (SPB < 100 mmHg) (%)	3.4	1.1		
Normotension (%)	78.7	79.6		
Hypertension (SPB > 160 mmHg) (%)	18.0	19.3		
DBP on ED admission (median mmHg)	80 (70–90)	80 (70–90)	0.923	15.3
Heart rate on ED admission (median min^− 1^)	80 (71–92)	80 (70–90)	0.515	16.5
Bradycardia (HR < 60/min) (%)	3.9	4.5		
Normocardia (%)	85.2	84.3		
Tachycardia (HR > 100/min) (%)	10.9	11.2		
SpO_2_ on ED admission (median %)	99 (97–100)	99 (97–100)	0.311	20.9
Hypoxia (SpO_2_ < 90%) (%)	0.7	3.5	0.021	
Hospitalized (%)	47.5	97.1	< 0.001	0.4
Hospitalization days (median)	0 (0–2)	3 (2–9)	< 0.001	6.2
ICU admission (%)	2.5	10.8	< 0.001	1.5
Neurosurgical Intervention (%)	0.1	6.9	< 0.001	1.4
Characteristics of the initial cerebral CT scan				
Time to first CT scan (median minutes)	82	61	< 0.001	8.4
Any trauma-related pathology (%)	7.7	86.6	< 0.001	4.3
Skull fracture (%)	12.8	35.7	< 0.001	30.8
Rotterdam CT score (median)	1 (1–2)	3 (2–3)	< 0.001	5.8
Marshall CT classification (%)			< 0.001	5.7
I	96.3	19.8		
II	3.4	68.1		
III	0.2	5.5		
IV	0.2	0		
VI	0	6.6		
Requirement for second CT scan (%)	1.8	27.0	< 0.001	3.5
Injury severity and Outcome				
H-AIS (median)	2 (1–2)	3 (3–4)	< 0.001	0
Injury Severity Score (median)	5 (3–8)	16 (10–25)	< 0.001	0.4
Glasgow Outcome Score (median)	5 (5–5)	5 (4–5)	< 0.001	1.5
Glasgow Outcome Score < 5 (%)	8.4	37.3	< 0.001	1.5
In-hospital mortality (%)	0.4	1.9	0.069	1.0

Anticoagulant use refers to the use of anticoagulant or antiplatelet drugs before the injury. Alcohol intoxication was assumed based on anamnestic data or by a blood alcohol concentration ≥ 0.05%. Drug Intoxication was determined based on anamnestic data or on positive drug screening results

*PEARL* ‘Pupils equal and reactive to light’, *EMS* Emergency Medical Services, *Office hours* Monday–Friday from 8 a.m. to 5 p.m., *GCS* Glasgow Coma Scale, *ED* Emergency Department, *SBP* systolic blood pressure, *DBP* diastolic blood pressure, *ICU* intensive care unit, *H-AIS* head abbreviated injury score

Table 2 shows the bivariate relationship between clinical predictor variables and discrepancies in initially assumed vs. actual injury severity after multiple imputation and accounting for clustering of the observations in two different trauma centers. Male gender, temporary postinjury loss of consciousness (LOC), amnesia, absence of pupils that are equal and reactive to light (PEARL), transport to hospital by emergency medical services (EMS), presence of hypoxia (SpO_2_ < 90%), as well as any GCS lower than 15 were associated with discrepancies between assumed and actual injury severity.

**Table 2:** Bivariate relationship between potential clinical predictor variables and discrepancies between initially assumed vs. actual injury severity, after multiple imputation and adjustment for clustering of the observations in two different trauma centers

	OR (95% CI)	*p* value
Clinical variables		
Male gender	2.1 (1.2–3.6)	0.011
Age ≥ 50 years	1.0 (0.5-2.0)	0.999
Age ≥ 65 years	0.9 (0.4–2.4)	0.886
Age (continuous)	1.0 (1.0–1.0)	0.958
Use of anticoagulants	1.0 (0.7–1.4)	0.947
Loss of consciousness	2.8 (1.8–4.3)	< 0.001
Amnesia	3.8 (1.3–10.6)	0.012
Nausea	1.1 (0.4–2.9)	0.807
Vomiting	2.4 (0.9–6.5)	0.085
Headache	1.2 (0.6–2.2)	0.614
Absence of PEARL	2.5 (2.2–2.8)	< 0.001
Alcohol Intoxication	0.8 (0.4–1.6)	0.623
Drugs Intoxication	1.5 (0.7–3.3)	0.286
Presented to hospital by EMS	2.1 (1.4-3.0)	< 0.001
Admission during office hours	0.9 (0.6–1.3)	0.494
Systolic blood pressure		
Normotension	Reference	
Hypotension	0.5 (0.1–3.3)	0.508
Hypertension	1.0 (0.4–2.4)	0.954
Heart rate		
Normocardia	Reference	
Bradycardia	1.1 (0.4–2.9)	0.835
Tachycardia	1.0 (0.7–1.4)	0.877
Hypoxia (SpO_2_ < 90%)	4.8 (1.6–14.6)	0.006
GCS at ED admission		
15	Reference	
14	3.3 (3.2–3.5)	< 0.001
13	7.0 (6.6–7.4)	< 0.001

Anticoagulant use refers to the use of anticoagulant or antiplatelet drugs before the injury. Alcohol intoxication was assumed based on anamnestic information or by a blood alcohol concentration ≥ 0.05%. Drug Intoxication was determined based on anamnestic information or on positive drug screening results. Normotension: systolic blood pressure 100–160 mmHg, Normocardia: hart rate 60–100 beats per min

*PEARL* ‘Pupils equal and reactive to light’, *EMS* Emergency Medical Services, *Office hours* Monday–Friday from 8 a.m. to 5 p.m., *GCS* Glasgow Coma Scale, *ED* Emergency Department

In the multivariable analysis (model A), an admission GCS score of 13 and hypoxia markedly increased the odds of being more severely injured than initially assessed (OR 6.2, 95% CI 3.8–9.9, and OR 5.4, 95% CI 1.2–23.4, respectively). LOC, absence of PEARL, transport to hospital by EMS, and a GCS score of 14 were associated with an approximately two to threefold increase in the odds of being more severely injured. Use of anticoagulants increased the odds by approximately 20% (Table 3).

**Table 3:** multivariable logistic regression showing the relationship between several clinical predictor variables and discrepancies between the initial assessment of injury severity and actual injury severity (Model A)

	OR (95% CI)	*p* value
Model A		
Use of anticoagulants	1.2 (1.1–1.3)	0.004
Loss of consciousness	2.3 (1.5–3.5)	< 0.001
Absence of PEARL	2.1 (1.6–2.7)	< 0.001
Presented to hospital by EMS	2.0 (1.7–2.4)	< 0.001
Hypoxia (SpO_2_ < 90%)	5.4 (1.2–23.4)	0.026
GCS at ED admission		
15	Reference	
14	2.7 (2.0-3.7)	< 0.001
13	6.2 (3.8–9.9)	< 0.001
Model B		
Use of anticoagulants	1.1 (0.9–1.3)	0.399
Loss of consciousness	1.9 (1.1–3.3)	0.032
Absence of PEARL	2.3 (1.4–3.6)	< 0.001
Presented to hospital by EMS	2.3 (1.4–3.6)	0.001
Hypoxia (SpO_2_ < 90%)	4.2 (1.5–11.8)	0.014
GCS at ED admission		
15	Reference	
14	1.7 (0.8–4.0)	0.190
13	2.1 (0.6–7.1)	0.213
Pathology on cerebral CT scan	66.2 (19.1–229)	< 0.001

All estimates are adjusted for the effects of the other independent variables in the model, and are corrected for clustering of the observations in two different trauma centers. Model B is additionally adjusted for the presence of trauma-related pathology on the CT scan. Anticoagulant use refers to the use of anticoagulant or antiplatelet drugs before the injury

*PEARL* ‘Pupils equal and reactive to light’, *EMS* Emergency Medical Services, *GCS* Glasgow Coma Scale, *ED* Emergency Department

After adjusting for the presence of trauma-related pathology on the cerebral CT scan (model B), LOC, absence of equal and reactive pupils, transport to hospital by EMS, and hypoxia remained significantly associated with the odds of being more severely injured than initially classified (Table 3).

## Discussion

We reviewed 808 patients with a clinical diagnosis of mild TBI admitted to two level I trauma centers in the Netherlands. 13% of these patients showed a discrepancy between the initial assessment of TBI severity based on the GCS, and the actual TBI severity as determined post hoc when all relevant information was available. A higher rate of hospitalization and longer hospital stay, a higher rate of admission to the intensive care unit, the increased need for neurosurgical interventions, and lower Glasgow Outcome Scale scores among patients with such a discrepancy underline that these patients are at risk for adverse outcomes and should be identified as early as possible.

Our data suggest that a GCS score < 15, temporary postinjury LOC, absence of PEARL, hypoxia, use of anticoagulants, or admission by EMS should alarm healthcare providers that the patient may be more severely injured than initially assessed. Such clinical variables are particularly important, because patients with an apparently mild injury may not receive the highest priority in busy emergency departments, and diagnostic tests that may reveal more serious injuries can be delayed for several hours, or may not be performed at all. In our patient population, approximately 70% of patients with mild TBI received a cerebral CT scan within 2 h after admission, and 5% of patients waited longer than 4 h until being scanned.

To our knowledge, studies specifically addressing clinical predictors of a discrepancy between initially classified TBI severity and actual injury severity in patients with apparently mild TBI are lacking. The previous studies, such as the ‘International Mission on Prognosis and Analysis of Clinical Trials’ (IMPACT) and ‘Corticoid Randomisation After Significant Head injury’ (CRASH) trials, have investigated the relationship between clinical variables and adverse outcomes in patients with predominantly moderate and/or severe TBI [14, 15]. However, outcomes as ‘death’ or ‘severe neurologic disability’ are uncommon in patients who present with an initial GCS between 13 and 15, and these studies provide only limited guidance to identify patients with apparently mild TBI who may actually be more severely injured.

Other studies have addressed the question which patients with mild TBI require a cerebral CT scan. The two most widely used scores are the Canadian CT Head Rule and the New Orleans Criteria, which have a very high sensitivity for detecting major intracranial injuries [16, 17]. Clearly, patients with apparently mild TBI who turn out to have relevant intracranial injuries are more severely injured than initially assumed. Yet, however, predictors of injuries on CT scans are not necessarily identical to clinical risk factors for the presence of injuries that are more severe than initially assumed in patients with apparently mild TBI. First, the studies in which the scores were developed limited inclusion to patients with LOC or amnesia, implying that patients without these signs are not at risk of having significant injuries. In contrast, the absence of these features does not exclude intracranial injuries [6, 18]. We, therefore, did not restrict inclusion to patients with LOC or amnesia, and in fact, in the present study, about 8% of the patients with a discrepancy between initially assumed and actual injury severity neither had LOC nor amnesia. Second, a normal initial CT scan does not exclude a clinically important injury, including delayed hemorrhage, which may still even occur up to several weeks after the initial trauma, or non-hemorrhagic injuries, such as traumatic axonal injury [19, 20]. In fact, 13% of the patients who turned out to be more severely injured than assumed in our patient population had an unremarkable initial CT scan.

This underlines that clinical variables may be a useful supplement to identify patients at risk. Not surprisingly, a part of the risk factors that we identified, such as absence of PEARL, LOC, and hypoxia, have previously been associated with adverse outcomes after TBI [2, 6, 14, 15]. Surprisingly, preinjury use of anticoagulant drugs was not a significant risk factor in the bivariate analysis, even though several previous studies suggest worse outcome after TBI in patients using anticoagulant drugs [21–23]. However, note that the bivariate analysis is subject to confounding and must be interpreted with care. After adjustment for other risk factors in the multivariable model, preinjury use of anticoagulants was, indeed, significantly associated with an approximately 20% higher odds to be more severely injured than initially assumed. Therefore, preinjury use of anticoagulants actually does need to be considered in the assessment of patients with apparently mild TBI.

To our knowledge, transport by EMS has previously not been studied as a potential risk factor for adverse outcomes. Interestingly, EMS transport was independently associated with a two-fold increase in the odds of a discrepancy between initially assumed and actual injury severity, and this also holds true after adjusting for the initial CT scan. Patients transported by EMS were comparable to patients who were not transported by EMS with respect to age, GCS scores, incidence of LOC, incidence of hypo-/hypertension, incidence of brady-/tachycardia, incidence of hypoxia, as well as incidence of use of anticoagulant drugs. Yet, these patients have an increased risk to be more severely injured than initially assessed. A likely explanation is that the variable EMS transport represents a surrogate for other (unobserved) variables that may be difficult to quantify, but which yet indicate more severe injury. Transport by EMS suggests that patients or bystanders perceived the accident as severe enough to warrant EMS activation, and that EMS personnel perceived the accident as severe enough to transport the patient by ambulance. It is not always clear in retrospect what the subjective or objective criteria were to transport a certain patient by ambulance, while EMS were not activated for another similar patient. Whatever the reasons may have been, our data suggest that patients who are presented by EMS should be closely monitored as they may be more severely injured than assumed.

## Limitations

We performed a retrospective study, with the inherent limitations of this research design. To minimize selection bias, all consecutive patients meeting the inclusion criteria were included. Nonetheless, the inclusion criteria may have led to some selection bias as we included only patients who received a CT scan. The decision to perform a CT scan was made using local protocols in the trauma centers, which are based on a guideline by the Dutch Society of Neurology [24]. According to this guideline, a CT scan is indicated for all patients with a GCS score < 15 at presentation, such that selection bias should only not have affected patients with a GCS score of 13 or 14. Patients with a GCS score of 15 also regularly receive a CT scan (609 patients in our study had a GCS score of 15), as the guideline defines a variety of other criteria (such as trauma mechanism, clinical signs of head injury, age, vomiting, temporary loss of consciousness, amnesia, or focal neurologic symptoms) which alone or in combination are used to determine the indication for a CT scan. Nonetheless, we may have missed patients in our analysis who presented to the ED with a trivial head trauma and a GCS score of 15.

To minimize information bias, we extracted and cross-checked the data across multiple data sources (electronic and hand-written medical records, and hospital’s trauma registration system) using a standardized data coding protocol. Nonetheless, we could not avoid missing data and, therefore, used multiple imputation to increase efficiency and to minimize bias which could occur in complete case analysis [25].

We compared the initial GCS scores at admission to H-AIS scores that were determined post hoc. While both scores are used to quantify injury severity, they do not measure exactly the same quantity and the comparison may at the first glance seem as comparing apples to oranges. However, we were specifically interested to assess discrepancies between the initial assessment of injury severity and the actual severity of the injury. The former is addressed by the GCS score at presentation, and the latter is characterized by the H-AIS score, which is scored by a specially trained and dedicated data manager of the trauma center when all information is available after diagnostics and treatment have been terminated. Hence, discrepancies between the initial GCS and H-AIS suggest that the patient was actually more severely injured than initially assessed, or that the patient deteriorated during hospital admission. Either way, these patients are at risk for under-treatment of injuries or complications.

Finally, we did not externally validate our results. Additional research is necessary to confirm whether the risk factors can also be used to identify patients at risk in hospitals that have a different case-mix.

## Conclusions

Head injured patients with apparently mild TBI, as defined by an initial GCS score of 13–15 at presentation, have a considerable risk of being more severely injured than initially classified. A GCS score of less than 15, LOC, absence of reactive and equal pupils, hypoxia, use of anticoagulant medication, and admission by EMS were associated with an increased risk. Such patients should be thoroughly monitored for signs of neurologic deterioration. An admission SpO_2_ < 90%, absence of equal and reactive pupils, transport by ambulance, and loss of consciousness are factors which indicate an increased risk, independently of the presence or absence of trauma-related pathology on the initial cerebral CT scan.
